# B vitamin blood concentrations and one-carbon metabolism polymorphisms in a sample of Italian women and men attending a unit of transfusion medicine: a cross-sectional study

**DOI:** 10.1007/s00394-020-02448-1

**Published:** 2020-12-29

**Authors:** Renata Bortolus, Francesca Filippini, Silvia Udali, Marianna Rinaldi, Sabrina Genesini, Giorgio Gandini, Martina Montagnana, Francesca Chiaffarino, Giuseppe Lippi, Patrizia Pattini, Gelinda De Grandi, Oliviero Olivieri, Fabio Parazzini, Simonetta Friso

**Affiliations:** 1 Office for Research Promotion, Verona University Hospital, Verona, Italy; 2 Unit of Internal Medicine, Department of Medicine, University of Verona School of Medicine, Verona, Italy; 3 Unit of Transfusion Medicine, Verona University Hospital, Verona, Italy; 4 Section of Clinical Biochemistry and Hematology, Verona University Hospital, Verona, Italy; 5 Department of Woman, Newborn and Child, Fondazione Istituto di Ricovero e Cura a Carattere Scientifico (IRCCS) Ca’ Granda Ospedale Maggiore Policlinico, Milan, Italy; 6 Department of Clinical Sciences and Community Health, Università Degli Studi di Milano, Milan, Italy

**Keywords:** Folate, Vitamin B12, Vitamin B6, One-carbon polymorphisms

## Abstract

**Purpose:**

To define blood status of folate, vitamin B12, vitamin B6, homocysteine, and major one-carbon metabolism-related polymorphisms in healthy, males and females blood donors, aged 18–65 years were evaluated. General characteristics and lifestyle factors were also investigated.

**Methods:**

An explorative cross-sectional study design was used to evaluate a sample of blood donors attending the Unit of Transfusion Medicine of the Verona University Hospital, Italy. From April 2016 to May 2018, 499 subjects were enrolled (255 men, 244 women of whom 155 of childbearing age). Major clinical characteristics including lifestyle and dietary habits, B vitamins and homocysteine were analyzed. The *MTHFR* 677 C>T, *cSHMT* 1420 C>T, *DHFR* 19 bp ins/del, *RFC1* 80 G>A polymorphisms were also determined.

**Results:**

Mean plasma concentrations of folate, vitamin B12, vitamin B6 and homocysteine were 14.2 nmol/L (95% CI 13.7–14.8), 271.9 pmol/L (95% CI 262.6–281.5), 51.0 nmol/L (95% CI 48.7–53.4) and 13.5 µmol/L (95% CI 13.1–14.0), respectively. Plasma folate, was adequate (> 15 nmol/L) in 44.7% of all subjects, 39.0% of males and 42.5% of women < 45 years. Similarly, vitamin B12 was adequate (> 350 pmol/L) in 25.1% of all subjects and in 20.3% of men ≥ 45 years. The rare allele frequencies were 0.21 for *MTHFR* 677TT, 0.11 for *cSHMT* 1420TT, 0.18 for *DHFR* 19 bp del/del, 0.20 for *RFC1* 80AA, and a gene–nutrient interaction was confirmed for folate concentrations according to *MTHFR* 677C>T and *DHFR* 19 bp del/del.

**Conclusion:**

An Italian sample of healthy blood donors shows that an adequate concentration of plasma folate and vitamin B12 is reached only in a limited percentage of subjects, thus encouraging consideration for specific public health strategies.

## Introduction

B vitamins are important to define the health status of a population because of their major role in affecting the risk of neural tube defects (NTDs) [1] and major chronic diseases [2–4]. The awareness of their precise blood concentrations is, therefore, a main issue to design public health policies [5] and even more so in a population without mandatory fortification especially if one considers the importance of establishing regional and national reference values. Only few data, however, are so far available from studies conducted in Italy on the average blood levels for B vitamins [6–8]. Recently, surveys conducted on Italians’ eating habits have shown that adherence to Mediterranean diet has decreased considerably over the last few years [9]. A study conducted in Central Italy among blood donors showed that total B vitamins levels were lower than those considered appropriate in men and women [7]. Similarly, a study conducted among pregnant women showed that the average red blood cells (RBCs) folate levels and plasma folate concentrations were at the lowest extreme of the range values at the beginning of pregnancy (8th–10th week of gestation) in women not reporting current use of folic acid supplementation [6].

Considering the interest of defining B vitamins intake with the diet for a clear definition of dietary policies together with the possible indication of supplement use, it is important to measure in a sample of healthy men and women, their plasma levels of folate and other major B vitamins, namely B12 and the active form of vitamin B6, pyridoxal 5′-phosphate (PLP), as well as that of homocysteine (tHcy). One-carbon metabolism-related genes should be also taken into account for their role in modulating vitamin concentrations and eventually the risk of diseases, in a gene–nutrient interaction manner [3, 8]. In this regard, the 5,10-methylenetetrahydrofolate reductase (*MTHFR*) 677C>T polymorphism is considered a paradigmatic example for gene–nutrient interactions affecting folate metabolism and potentially the disease risk [8, 10]. As for other polymorphic sites in folate-metabolism-related genes, the insertion/deletion (19 bp ins/del) at dihydrofolate reductase (*DHFR*) gene [11] has also an important role for biochemical modification of folate-related tHcy metabolism [12]. Furthermore, the reduced folate carrier 1 (*RFC1*) gene 80 G>A polymorphism is of considerable interest for its function in guaranteeing adequate folate absorption [13]. A functional polymorphism described in *cSHMT* gene, the 1420 C>T, affects the ability of the enzyme to pursue either the nucleotide synthesis or the methyl group provision within the folate-related one-carbon metabolism [14]. The allele frequency for those polymorphisms is yet mostly unknown in the general population though of interest for their link with B vitamins.

The primary objective of this study was to measure, in a sample of men and women ranging 18–65 years of age and attending a Unit of Transfusion Medicine, their nutritional status by the assessment of plasma concentrations of folate, vitamin B12, vitamin B6 and tHcy. The main determinants of B vitamins were also considered, as were the genotyping for *MTHFR* 677 C>T, *cSHMT* 1420 C>T, *DHFR* 19 bp ins/del, *RFC1* 80 G>A.

## Subjects and methods

### Study design and participants

From April 2016 to May 2018, 551 healthy blood donors, consecutively attending the Unit of Transfusion Medicine of the Verona University Hospital, Italy were asked to participate to this cross-sectional study, of them 538 subjects (97.6%) agreed. In Italy blood donors are allowed to donate whole blood four times per year as a maximum, and two times per year for women in childbearing age. In the Unit of Transfusion Medicine of the Verona University Hospital donors are only admitted periodically for a mean number of 2.1 blood donations per year. Exclusion criteria were vitamins supplementation during the last 2 months before the blood sample withdrawal. Among all the participants, 37 were excluded after enrolment into the study, because they subsequently reported supplementation with folic acid and/or other vitamins during the 2 months prior to the blood sample withdrawal. Blood samples values from two other subjects were not considered for the data analyses due to pre-analytical technical problems. Overall, 499 subjects were enclosed into the study (255 men, 244 women: of these 155 were of childbearing age 18–44 years).

During the visit for blood donation enrolment, each eligible subject, after a detailed explanation of the study by the researcher, was invited to participate. After giving written informed consent, each participant was interviewed about his/her general characteristics, medical history and current therapy. Lifestyle and dietary habits including alcohol and smoking habits, fruits and vegetables consumption were also recorded. For fruits and vegetables one portion was defined as 150 g and 100 g, respectively.

### Laboratory parameters

Venous whole blood samples were withdrawn after an overnight fasting into Vacutainer® tubes containing ethylenediaminetetraacetic acid (EDTA) as anticoagulant to obtain plasma or without anticoagulant to obtain serum, as appropriate for the specific analyses.

After centrifugation at 1.500 g for 10 min at room temperature, plasma was separated, stored in aliquots and kept frozen at − 70 °C until measurements. Plasma folate and vitamin B12 concentrations were measured by an automated chemiluminescence method on Cobas e801 (Roche Diagnostics, Mannheim, Germany). Vitamin B6 was measured as PLP, its active form, by high-performance liquid chromatography (HPLC) with fluorometric detection [4]. Plasma tHcy concentrations were determined by an automated chemiluminescence method on ADVIA Centaur (Siemens Diagnostics) [3].

Venous blood was withdrawn from each subject into Vacutainer® tubes containing EDTA as anticoagulant after an overnight fast and stored at − 20 °C until analysis for genotyping, DNA was extracted from peripheral blood mononuclear cells (PBMCs) by Wizard Genomic DNA Purification Kit (Promega Corporation, Fitchburg, WI, USA). Genotyping for one-carbon-related polymorphisms (*MTHFR* 677 C>T, *cSHMT* 1420 C>T, *DHFR* 19 bp ins/del, *RFC1* 80G>A) were analysed by different methods: *MTHFR* 677C>T (rs1801133) [15], *cSHMT* 1420C>T (rs1979277) [16], *DHFR* 19 bp ins/del (rs70991108) [17], *RFC1* 80G>A (rs1051266) [13] and by PCR followed by restriction fragment length polymorphism assays. Data on genotyping were not determined due to poor yield for one sample.

The adequate status of plasma folate, vitamin B12 and tHcy was defined according to previous reports by others [2, 7] as follows: folate > 15 nmol/L, vitamin B12 > 350 pmol/L and tHcy < 10 µmol/L. The concentration of plasma PLP > 35 nmol/L [4], was defined adequate [18]. Considering the significance of B vitamins in women or men of different age, data were analysed according to different age and gender categories. In women of childbearing age the risk of NTDs in pregnancy was modelled by Daly et al. [19]: the risk increased when maternal plasma folate concentration was less than 15.9 nmol/L. Chen MY et al. [20] indicated that optimal RBC folate concentration was associated with a mean plasma folate concentration of 25.5 nmol/L: when this cut-off was considered, the risk increased for maternal plasma folate concentration < 25.5 nmol/L.

### Statistical analysis

The data were collected in a specific database after a review for completeness, consistency and plausibility.

The primary objective of this study was to estimate the frequency of adequate plasma folate concentrations (> 15 nmol/L) in the whole study population and separately among men and women. We foresaw to include into the study about 250 women and 250 men. With this sample size we were able to obtain an estimate of adequate plasma folate concentration with narrow 95% confidence interval (CI). For example, considering separately men and women, the expected 95% CI were, respectively, 43.6–56.4 if the adequate plasma folate concentrations were 50% or 69.4–80.4 if the value was 70%, a frequency close to that reported by Zappacosta et al. [7].

Continuous variables were calculated by mean ± standard deviation (SD), while logarithmic transformation was used for non-normal distribution variable and geometric means and CIs were used, as appropriate. Categorical variables were presented by calculating absolute frequency and percentage. The 95% CIs of the mean and proportion were provided to assess the precision of the estimates. Genetic data were analysed to evaluate the frequency of each genotype in the population studied after evaluating the Hardy Weinberg equilibrium. Considering the well-known role of gene–nutrient interactions within folate and B vitamin metabolism, separate analysis was performed according to plasma concentrations of folate by considering the median values, according to *MTHFR* 677 C>T, *cSHMT* 1420 C>T, *DHFR* 19 bp ins/del, *RFC1* 80G>A, genotypes.

Categorical variables were compared using the Pearson or Mantel–Haenszel Chi-square, as appropriate. Continuous variables were analyzed using analysis of variance, after logarithmic transformation if needed, or Kruskal–Wallis test when appropriate. We considered a two-tailed *P* value of < 0.05 to be significant. Odds Ratios (ORs) for inadequate status of plasma folate, vitamin B12, vitamin B6 and tHcy according to sociodemographic and general characteristics were computed. To take into account potential confounding factors, we used unconditional multiple logistic regression, with maximum likelihood fitting including in the model terms for gender, age, education, body mass index (BMI), smoking, alcohol drinking, fruits and vegetables consumption and physical activity. All the analyses were performed using the SAS software, version 9.4 (SAS Institute, Inc, Cary, NC, USA).

## Results

### Characteristics of the study population

**Fig. 1:**
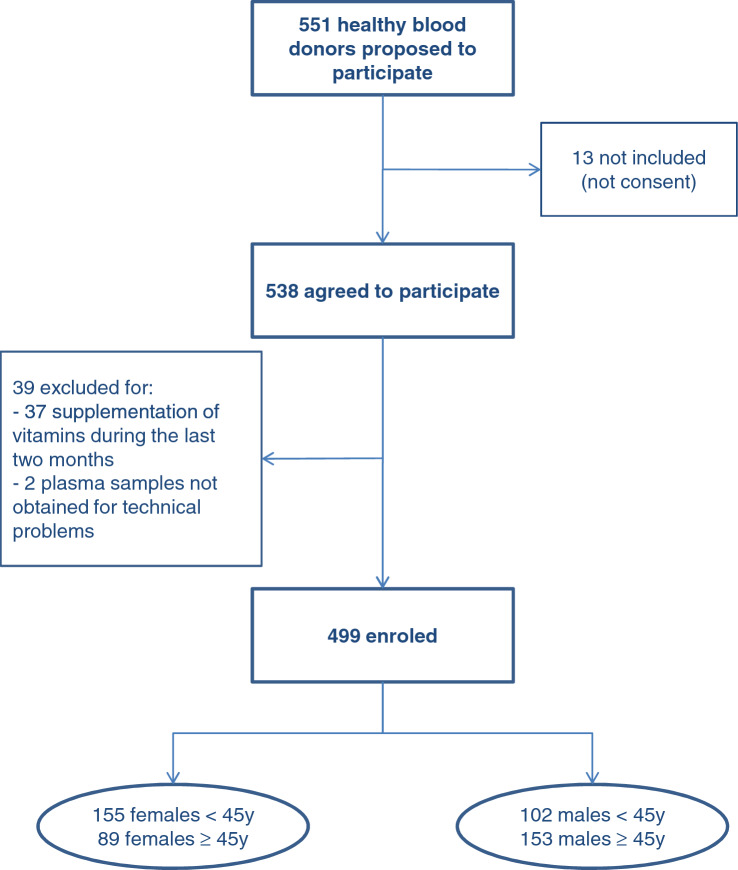
Flow diagram of the study design

The flow diagram of the study is reported in Fig. 1. Sociodemographic and general characteristics of the subjects according to age and gender are shown in Table 1.

**Table 1:** Sociodemographic and general characteristics of the subjects according to age and gender

	Total *n* (%)	Females < 45 years *n* (%)	Females ≥ 45 years *n* (%)	*P* value*	Males < 45 years *n* (%)	Males ≥ 45 years *n* (%)	*P* value*
Age (years)							
≤ 34	124 (24.9)	94 (60.7)			30 (29.4)		
35–44	133 (26.7)	61 (39.4)			72 (70.6)		
45–54	164 (32.9)		73 (82.0)			91 (59.5)	
≥ 55	78 (15.6)		16 (18.0)			62 (40.5)	
Education (years)				0.0002			< 0.0001
≤ 8	80 (16.0)	9 (5.8)	21 (23.6)		6 (5.9)	44 (28.8)	
9–15	301 (60.3)	91 (58.7)	46 (51.7)		72 (70.6)	92 (60.1)	
≥ 16	118 (23.7)	55 (35.5)	22 (24.7)		24 (23.5)	17 (11.1)	
BMI (kg/m^2^)				0.0536			0.1909
Underweight	12 (2.4)	10 (6.5)	1 (1.1)		0	1 (0.7)	
Normalweight	328 (65.7)	111 (71.6)	74 (83.2)		62 (60.8)	81 (52.9)	
Overweight	127 (25.5)	25 (16.1)	13 (14.6)		35 (34.3)	54 (35.3)	
Obesity	32 (6.4)	9 (5.8)	1 (1.1)		5 (4.9)	17 (11.1)	
Current smoking				0.5678			0.003
No	424 (85.0)	128 (82.6)	76 (85.4)		80 (78.4)	140 (91.5)	
Yes	75 (15.0)	27 (17.4)	13 (14.6)		22 (21.6)	13 (8.5)	
Alcohol intake				0.5218			0.03
No	120 (24.1)	58 (37.4)	37 (41.6)		5 (4.9)	20 (13.1)	
Yes	379 (76.0)	97 (62.6)	52 (58.4)		97 (95.1)	133 (86.9)	
Fruits				0.0709			0.1615
< 1 portion/day	138 (27.7)	46 (29.7)	16 (18.0)		37 (36.3)	39 (25.5)	
1–2 portion/day	278 (55.7)	88 (56.8)	54 (60.7)		48 (47.1)	88 (57.5)	
> 2 portion/day	83 (16.3)	21 (13.6)	19 (21.4)		17 (16.7)	26 (17.0)	
Vegetables				0.0083			0.7859
< 1 portion/day	55 (11.0)	15 (9.7)	1 (1.1)		15 (14.7)	24 (15.7)	
1–2 portion/day	370 (74.2)	101 (65.2)	72 (80.9)		78 (76.5)	119 (77.8)	
> 2 portion/day	74 (14.8)	39 (25.2)	16 (18.0)		9 (8.8)	10 (6.5)	
Physical activity				0.8962			0.7895
< 2 h/week	200 (40.1)	68 (43.9)	38 (42.7)		40 (39.2)	54 (35.3)	
2–5 h/week	197 (39.5)	66 (42.6)	37 (41.6)		37 (36.3)	57 (37.3)	
> 5 h/week	102 (20.4)	21 (13.6)	14 (15.7)		25 (24.5)	42 (27.5)	

*P value were obtained by Chi-squared test for the comparison within each gender group, i.e., among females and males, divided by age either < 45 years or ≥ 45 years. Underweight and normal weight were considered together

### Biochemical parameters

In the study population mean plasma folate concentrations were 14.2 nmol/L (95% CIs 13.7–14.8), plasma vitamin B12 concentrations were 271.9 pmol/L (95% CIs 262.6–281.5) and mean plasma vitamin B6 51.0 nmol/L (95% CIs 48.7–53.4); plasma tHcy concentrations were 13.5 µmol/L (95% CIs 13.1–14.0). Females showed higher plasma folate concentrations as compared to males, while males had significantly higher plasma concentrations of vitamin B6 and tHcy (Table 2). As shown in Table 2, females < 45 years of age had lower plasma folate concentration compared to females of age ≥ 45 years, as expected, while males < 45 years had higher plasma concentrations of vitamin B12, B6 and lower tHcy as compared to males  ≥ 45 years.

**Table 2:** Mean plasma concentrations and prevalence of subjects with adequate B vitamins and tHcy according to gender and age

	Total *n* = 499	Females *n* = 244	Males *n* = 255	*P* value*	Females < 45 years *n* = 155	Females ≥ 45 years *n* = 89	*P* value*	Males < 45 years *n* = 102	Males ≥ 45 years *n* = 153	*P* value*
Folate (nmol/L)	14.2^a^ (13.7–14.8)	14.8 (14.0–15.7)	13.7 (13.0–14.4)	0.0346	14.1 (13.1–15.2)	16.1 (14.7–17.7)	0.0256	13.7 (12.6–14.8)	13.6 (12.8–14.6)	0.9624
*n* (%) > 15 nmol/L^b^	220 (44.7)	116 (47.5)	104 (40.8)	0.1286	65 (42.5)	51 (58.0)	0.0206**	39 (39.0)	65 (43.1)	0.5241**
Vitamin B12 (pmol/L)	271.9^a^ (262.6–281.5)	278.5 (264.5–293.2)	265.7 (253.5–278.5)	0.1839	273.4 (254.8–293.4)	287.6 (267.9–308.7)	0.3539	299.1 (280.4–319.0)	245.8 (230.8–261.7)	< 0.0001
*n* (%) > 350 pmol/L^b^	125 (25.1)	64 (26.2)	61 (23.9)	0.5519	40 (25.8)	24 (27.0)	0.8428**	30 (29.7)	31 (20.3)	0.0847**
Vitamin B6 (nmol/L)	51.0^a^ (48.7–53.4)	48.3 (45.0–51.9)	53.8 (50.7–57.0)	0.0230	49.6 (45.2–54.4)	46.2 (41.6–51.4)	0.3467	62.2 (56.5–68.4)	48.8 (45.4–52.5)	0.0001
*n* (%) > 35 nmol/L^b^	387 (77.7)	178 (73.0)	209 (82.0)	0.0158	119 (77.3)	59 (66.3)	0.0625**	93 (91.2)	116 (75.8)	0.0018**
tHcy (µmol/L)	13.5^a^ (13.1–14.0)	11.8 (11.3–12.3)	15.4 (14.8–16.0)	< 0.0001	11.6 (11.0–12.3)	12.2 (11.4–13)	0.3067	14.5 (13.7–15.3)	16.1 (15.2–17.0)	0.0108
*n* (%) < 10 μmol/L^b^	80 (16.1)	61 (25.0)	19 (7.5)	< 0.0001	41 (26.5)	20 (22.5)	0.4895**	9 (8.8)	10 (6.7)	0.5050**

^a^Values are given as means and CIs

^b^Adequate concentrations

*P value ≤ 0.05 was considered statistically significant

**P value were obtained by Chi-squared test for the comparison within each gender group, i.e., among females and males, divided by age either < 45 years or ≥ 45 years

Adequate plasma folate concentrations, defined for values > 15 nmol/L, were observed in 44.7% of blood donors. Adequate vitamin B12 concentrations, as defined for values > 350 pmol/L, were observed in 25.1% of total blood donors and in 20.3% of men ≥ 45 years. Adequate vitamin B6 concentrations, defined for values > 35 nmol/L, were observed in 77.7% of total blood donors (Table 2).

Among females and males < 45 years, 42.5% and 39.0% showed adequate plasma folate concentrations, respectively (Table 2). When the cut-off ≥ 15.9 nmol/L was considered for women at childbearing age [19], 39.9% showed adequate concentrations. Chen et al. [20] indicated that optimal RBC folate concentration was associated with a mean plasma folate concentration of 25.5 nmol/L: when this cut-off was considered, 11.8% showed adequate concentrations. Total plasma tHcy concentrations defined as ideal if < 10 µmol/L, were observed in 16.1% of total blood donors (Table 2). Concentrations of tHcy inversely correlated, as expected, with plasma levels of folate and vitamin B12 (data not shown).

In Table 3, the age ≥ 35 years and the intake of at least two portions/day of fruits and vegetables were associated with adequate concentrations of plasma folate. Regarding BMI only overweight subjects were related to inadequate concentration of folate.

**Table 3:** Odds ratios for inadequate status of B vitamins and tHcy according to general characteristics and lifestyle factors

	Folate ≤ 15 nmol/L^a^	Vitamin B12 ≤ 350 pmol/L^a^	Vitamin B6 ≤ 35 nmol/L^a^	tHcy > 10 µmol/L^a^
	OR (95% CI)*	OR (95% CI)*	OR (95% CI)*	OR (95% CI)*
Gender^b^				
Female	0.76 (0.48–1.18)	0.88 (0.53–1.43)	2.54 (1.47–4.40)	0.31 (0.16–0.58)
Age (years)^b^				
35–44	0.42 (0.22–0.81)	0.90 (0.46–1.76)	1.00 (0.43–2.40)	0.96 (0.94–2.14)
45–54	0.59 (0.33–1.07)	1.55 (0.83–2.90)	2.02 (1.00–4.12)	1.12 (0.56–2.26)
≥ 55	0.44 (0.23–0.85)	1.25 (0.63–2.47)	2.86 (1.31–6.26)	1.45 (0.63–3.33)
Education (years)^b^				
9–15	0.84 (0.47–1.48)	1.16 (0.62–2.14)	0.56 (0.31–1.00)	1.19 (0.54–2.64)
≥ 16	0.54 (0.28–1.06)	1.15 (0.56–2.39)	0.54 (0.26–1.14)	1.04 (0.43–2.54)
BMI (kg/m^2^)^b^				
Underweight	1.10 (0.30–4.03)	2.05 (0.43–9.85)	1.19 (0.28–4.99)	1.06 (0.26–4.30)
Overweight	1.76 (1.10–2.83)	1.33 (0.79–2.25)	1.39 (0.82–2.38)	1.22 (0.62–2.38)
Obesity	0.69 (0.32–1.51)	0.65 (0.29–1.43)	1.02 (0.40–2.60)	1.11 (0.35–3.51)
Current smoker^b^				
Yes	1.30 (0.76–2.22)	1.16 (0.64–2.10)	1.79 (1.00–3.20)	1.35 (0.64–2.85)
Alcohol drinking^b^				
Yes	0.96 (0.59–1.56)	0.90 (0.53–1.51)	0.86 (0.51–1.46)	1.19 (0.67–2.11)
Fruits and vegetables^b^				
2 portion/day	0.36 (0.22–0.57)	0.98 (0.60–1.60)	0.65 (039–1.08)	0.63 (0.32–1.20)
≥ 3 portion/day	0.27 (0.15–0.46)	1.00 (0.58–1.89)	0.35 (0.18–0.71)	0.49 (0.24–1.00)
Physical activity^b^				
2–5 h/week	0.80 (0.52–1.24)	1.17 (0.73–1.89)	0.85 (0.52–1.39)	0.95 (0.55–1.67)
> 5 h/week	0.67 (0.39–1.14)	0.75 (0.43–1.30)	0.66 (0.34–1.28)	1.06 (0.51–2.19)

*OR* odds ratio

*Multivariate estimates including in turn term for gender, age, education, BMI, smoking, alcohol drinking, fruits and vegetables intake, physical activity

^a^Inadequate concentrations

^b^Reference categories: male (gender), ≤ 34 years (age), < 8 years (education), normal weight (BMI), no (current smoker), no (alcohol drinking), < 1 portion/day (fruits and vegetables), < 2 h/week (physical activity)

No significant differences emerged in vitamin B12 for selected characteristics, while female gender and age ≥ 45 years were associated with inadequate concentration of vitamin B6. Moreover, intake of ≥ 3 portion/day of fruits and vegetables was associated with adequate concentrations of vitamin B6, while female gender was related to adequate concentration of tHcy (Table 3). No significant differences emerged in B vitamins for physical activity (Table 3).

### One-carbon metabolism-related polymorphisms

The homozygous mutant allele frequencies were 0.21 for the *MTHFR* 677TT, 0.11 for the *cSHMT* 1420TT, 0.18 for the *DHFR* 19 bp del/del and 0.20 for the *RFC1* 80AA.

Mean plasma folate concentrations were the lowest (13.0, 95% CIs 11.9–14.2) and tHcy the highest (15.7, 95% CIs 14.4–17.0) for carriers of the *MTHFR* 677TT genotype and significantly different for the comparison of the *MTHFR* 677TT vs. the 677CC. The carriers of the *DHFR* 19 bp del/del mutant allele showed lower concentrations of vitamin B12 as compared to the *DHFR* 19 bp ins/ins genotypes. The carriers of the *RFC1* 80AA and *cSHMT* 1420TT mutant genotypes did not show differences as compared to their corresponding wild-type genotypes for any of the B vitamins concentrations. No differences were also observed, in plasma concentrations of vitamin B6, for any of the polymorphisms analysed (data not shown).

As expected, the *MTHFR* 677C >T genotype homozygous mutants (677TT) showed the highest tHcy levels (18.5 µmol/L) with a significant difference between 677TT group according to plasma concentrations of folate either below or above the median value (18.5 vs. 12.9 µmol/L, *P* < 0.0001) (Table 4).

**Table 4:** Mean plasma concentrations of B vitamins and tHcy according to folate status and *MTHFR* 677 C > T genotype

	*MTHFR* 677CC *n* = 144			*MTHFR* 677TT *n* = 102		
	Low folate *n* = 64	High folate *n* = 79	*P* value*	Low folate *n* = 57	High folate *n* = 42	*P* value*
Vitamin B_12_ (pmol/L)	250.0 (226.9–275.5)	290.8 (270.1–313.1)	0.0131	232.9 (209.3–259.2)	267.1 (235.0–303.5)	0.1012
Vitamin B_6_ (nmol/L)	41.5 (36.8–46.7)	54.8 (49.8–60.2)	0.0003	41.8 (37.5–46.7)	53.9 (45.2–64.2)	0.0117
tHcy (µmol/L)	13.4 (12.5–14.5)	11.7 (11.1–12.4)	0.0034	18.5 (16.7–20.6)	12.9 (11.5–14.4)	0.0001

In some cases, the sum does not add up to the total because of missing value

Values are given as means and CIs

^*^P value ≤ 0.05 was considered statistically significant; *P* value was calculated for the comparison of the groups at low vs high plasma folate concentrations defined according to the median value levels (14.2 nmol/L)

Comparisons for tHcy according to the *RFC1* 80G > A genotypes showed that those with the lower levels of folate had higher levels of tHcy and there was significant difference within the *RFC1* 80GG and *RFC1* 80AA groups according to either low vs. high levels of folate concentrations (Table 5). Similar results were also observed for the *cSHMT* 1420 C > T and the *DHFR* 19 bp ins/del (Table 5).

**Table 5:** Mean plasma concentrations of B vitamins and tHcy according to folate status and *cSHMT* 1420 C > T, *DHFR* 19 bp ins/del and *RFC1* 80 G > A genotypes

	*cSHMT 1420CC* *n* = *250*			*cSHMT 1420TT n* = *51*		
	Low folate (< median value) *n* = 130	High folate (> median value) *n* = 118	*P* value*	Low folate (< median value) *n* = 30	High folate (> median value) *n* = 21	*P* value*
Vitamin B_12_ (pmol/L)	252.7 (236.5–269.9)	296.2 (275.7–318.2)	0.0014	236.9 (203.8–275.4)	314.1 (276.2–357.1)	0.0080
Vitamin B_6_ (nmol//L)	44.0 (40.4–48.0)	57.7 (52.5–63.5)	< 0.0001	40.5 (34.5–47.6)	52.5 (42.1–65.5)	0.0502
tHcy (µmol/L)	15.2 (14.2–16.1)	11.7 (11.1–12.4)	< 0.0001	15.5 (13.6–17.5)	12.3 (11.0–13.7)	0.0105

	*DHFR* 19 bp ins/ins *n* = 178			*DHFR* 19 bp del/del *n* = 83		
	Low folate (< median value) *n* = 83	High folate (> median value) *n* = 95	*P* value*	Low folate (< median value) *n* = 40	High folate (> median value) *n* = 41	*P* value*
Vitamin B_12_ (pmol/L)	259.7 (238.6–282.8)	308.9 (287.0–332.4)	0.0024	252.1 (225.9–281.5)	264.7 (229.7–303.9)	0.5977
Vitamin B_6_ (nmol//L)	45.3 (40.9–50.1)	52.4 (47.9–57.3)	0.0332	45.7 (38.8–53.8)	65.5 (53.9–79.5)	0.0056
tHcy (µmol/L)	15.5 (14.4–16.7)	11.7 (11.1–12.3)	< 0.0001	15.1 (13.7–16.7)	12.8 (11.7–13.9)	0.0123

	*RFC1* 80 GG *n* = 143			*RFC1* 80AA *n* = 96		
	Low folate (< median value) *n* = 72	High folate (> median value) *n* = 69	*P* value*	Low folate (< median value) *n* = 55	High folate (> median value) *n* = 40	*P* value*
Vitamin B_12_ (pmol/L)	251.7 (231.0–274.3)	296.2 (270.7–324.1)	0.0100	262.8 (237.4–290.8)	291.8 (256.7–331.6)	0.1950
Vitamin B_6_ (nmol//L)	42.1 (38.0–46.7)	58.1 (51.8–65.3)	< 0.0001	47.3 (41.4–54.1)	64.4 (55.1–75.3)	0.0034
tHcy (µmol/L)	16.2 (14.9–17.5)	11.8 (10.9–12.7)	< 0.0001	15.5 (14.1–17.1)	11.6 (10.7–12.7)	< 0.0001

Values are given as means and CIs

^*^P value ≤ 0.05 was considered statistically significant; *P* value was calculated for the comparison of the groups at low vs. high plasma folate concentrations defined according to the median value levels (14.2 nmol/L)

## Discussion

This study presents information on blood concentrations of folate, vitamin B12, vitamin B6, tHcy and related polymorphisms frequencies in a sample of healthy, Italian women and men aged 18–65 years, attending a Unit of Transfusion Medicine in Northern Italy.

The sample shows that an adequate concentration of plasma folate and vitamin B12 is reached only in a limited percentage of subjects, and a gene–nutrient interaction is confirmed for folate concentrations according to *MTHFR* 677 C > T and *DHFR* 19 bp del/del.

A previous study already showed a moderate impairment of B vitamins status in the area of Central-Southern Italy [7]. The results of our study also show no substantial differences for the percentage of subjects with adequate folate status among males and females. Interestingly, this study highlights that a large portion of healthy blood donors has an impaired vitamin B12 status and an inadequate status of tHcy especially in males, confirming previous studies [7, 21]. Results from this study, furthermore, confirm the higher plasma concentrations of folate among subjects with greater consumption of fruits and vegetables [7, 22].

Several studies evaluated folate, vitamin B12 and B6 status, and tHcy concentrations, in research with a rather diversified study design, where the cut-off used, the presence of food fortification, the lifestyle and dietary habits of population make the comparison among studies difficult. We referred to the cut-off values according to Dhonukshe-Rutten et al. [2] and Daly et al. [19] for the prevention of cardiovascular diseases and NTDs, with the knowledge that such values do not necessarily reflect a nutritional deficiency but rather a cut-off value associated to the prevention of these events [2–5, 8, 19].

A systematic review evaluating more than sixty studies, showed the B vitamin status was inadequate in several European countries [2]. In the IMMIDIET Project [23], among Italian and British women of childbearing age, the 66.7% and 22.1% of, respectively, showed folate serum concentrations < 15 nmol/L, similarly to the results of the present study.

In our study there was no association of folate status with either smoking and alcohol intake. Regarding smoking habit, a previous research in Italian blood donors showed no association when analyzed folate concentrations in blood [24], but low serum folate was related with smoking in others [23, 25]. Moreover, studies that evaluated the effects of alcohol consumption on serum folate indicated that the relationship could depend on the type of alcoholic beverage consumed [26].

An association was observed between plasma folate and BMI that was statistically significant only in overweight persons. As for folate status in obese women of childbearing age [27] and the impact of BMI on plasma folate in adults [28] there are few and conflicting results.

In the United States, age and dietary supplements had the greatest effects on prevalence estimates of low folate concentrations during the pre-fortification period [29, 30].

As for women in childbearing age, the present study shows adequate levels of plasma folate in 39.9% of women (cut-off  ≥ 15.9 nmol/L [19]). In a previous study by Zappacosta et al. [7], when the cut-off of RBCs folate used was  ≥ 906 nmol/L-1 [19], no women of childbearing age had adequate levels (7). These results were confirmed in a Swedish population [31].

Fayyaz et al. [32] described folate, vitamin B12 and B6 status in the Alberta Pregnancy Outcomes and Nutrition cohort. Folate deficiency was rare (3%), as well as vitamin B12 and B6 (< 1%), but 24% of women in their first trimester of pregnancy had suboptimal RCFs concentrations if considered for values < 906 nmol/L [32].

Our results indicate inadequate concentrations of vitamin B12 in most blood donors, especially in men ≥ 45 years and are similar to those by Sofi et al. [21], but not others [25, 33–35].

Regarding vitamin B6, adequate concentrations were observed in 77.7% of total blood donors in the present study, with significant differences for gender and age. Studies that evaluated the status of vitamin B6 were conducted mainly in the elderly population [36] and in relationship to cognitive functions [37, 38]. Determinants of PLP concentrations were also investigated [39–41].

A moderate reduction of PLP (36.3 nmol/L) was found to be inversely related to major markers of inflammation and independently associated with increased coronary artery disease risk in an Italian cohort and in the US participants from the population-based Framingham Heart Study cohort [42], showing that a mild vitamin B6 impairment is associated to major disease risk.

B vitamins have been mostly related to the risk of major chronic diseases in relationship to their role as determinants of tHcy [3, 4]. As consistently reported in most of the studies [2, 43], tHcy levels were inversely correlated with folate and serum vitamin B12 concentrations. As for the plasma concentrations of tHcy in the present study we observed higher levels of tHcy in men than in women, according to previous reports [2], where the authors observed that tHcy levels differed to a great extent between countries: from 7.1 to 14.8 µmol/L. Also in Italy the concentrations varied greatly [8, 21]. In our study, unlike others [44, 45], no significant differences emerged in tHcy for lifestyle factors.

As for the one-carbon metabolism-related genotypes, as expected the frequencies observed in the present study were similar to those reported in other European populations [3, 11–13, 16, 17]. The *MTHFR* 677C>T genotype was related to higher concentrations of tHcy according to folate status as previously reported [3], but also by other genotypes such as the *cSHMT* 1420C>T, the *DHFR* 19 bp ins/del and the *RFC1* 80G>A when data were analysed according to the median folate status. This finding is important to highlight the role of a gene–nutrient interaction with B vitamins for those common polymorphisms.

The study has some weaknesses and strengths. This cross-sectional study is based on a small and possibly specific population, perhaps not representative of the Italian healthy adult population. We evaluated blood donors, considered a healthier group than the majority of people and results expected in the general population could be different. Nevertheless, we approached this potential bias by balancing the age and sex groups.

Moreover, elderly people are not eligible for blood donation and, therefore, excluded.

In our study we assessed the folate status of subjects by measuring serum folate concentrations. The World Health Organization released guidelines that recommend RBCs folate concentrations above 906 nmol/L, using the microbiologic assay, for optimal protection from NTD-affected pregnancy [46]. The RBCs folate concentration should be used to evaluate such risk among different populations of childbearing age women. However, many population-based surveys do not measure RBCs folate concentrations and this methodology is not widely available in all countries, including Italy. Furthermore, some authors consider the serum assay, when available, an efficient marker to define folate status [47–49].

Finally, the cut-off used, the presence of mandatory food fortification, lifestyle and dietary habits of population investigated in different contexts and subgroups, make the comparison difficult.

Among the strengths of this study design, there is the fact of having evaluated people living in a European country without mandatory fortification, having also analysed the allele frequency of common genetic variants related to B-vitamins metabolism and, therefore, highlighting gene–nutrient interaction relationships with possible implication for nutritional policies in our population.

Considering the role of folate status in the prevention of birth defects and major chronic diseases, diversified intervention strategies should be implemented. Specifically, for women of childbearing age, although the prevalence of preconception folic acid supplementation in Europe has increased after many years of delivered recommendations, a large number of women still do not follow the recommendations and a lot of them start the supplementation too late [50].

Regarding vitamin B12, no associations were found between serum levels of vitamin B12 and selected characteristics considered. This makes the reasons of the inadequate B12 status in blood donors difficult to understand, but an important priority to be explored.

The results of the present cross-sectional study confirm inadequate concentrations of serum folate, vitamin B12 and tHcy in Italian women and men between 18 and 65 years. Public health strategies and integrated intervention programs should be undertaken and implemented, especially in subgroups of population with special needs, such as women of childbearing age.

## Abbreviations

BMI: Body mass index
CI: Confidence interval
*cSHMT*: Cytosolic serine hydroxymethyltransferase
*DHFR*: Dihydrofolate reductase
EDTA: Ethylenediaminetetraacetic acid
HPLC: High-performance liquid chromatography
*MTHFR*: Methylenetetrahydrofolate reductase
NTDs: Neural tube defects
OR: Odds ratio
PBMCs: Peripheral blood mononuclear cells
PLP: Pyridoxal 5′-phosphate
RBCs: Red blood cells
RCFs: Red cell folates
*RFC1*: Reduced folate carrier 1
SD: Standard deviation
tHcy: Total homocysteine
